# Salt Inducible Kinase Signaling Networks: Implications for Acute Kidney Injury and Therapeutic Potential

**DOI:** 10.3390/ijms20133219

**Published:** 2019-06-30

**Authors:** Mary Taub

**Affiliations:** Biochemistry Dept, Jacobs School of Medicine and Biomedical Sciences, University at Buffalo, 955 Main Street Suite 4102, Buffalo, NY 14203, USA; biochtau@buffalo.edu

**Keywords:** kidney proximal tubule, acute kidney failure, signal transduction, transcription, CREB Regulated Transcriptional Coactivators (CRTC), cAMP Regulatory Element Binding Protein (CREB), Salt Inducible Kinase (SIK), Class IIa Histone Deacetylases (HDAC)

## Abstract

A number of signal transduction pathways are activated during Acute Kidney Injury (AKI). Of particular interest is the Salt Inducible Kinase (SIK) signaling network, and its effects on the Renal Proximal Tubule (RPT), one of the primary targets of injury in AKI. The SIK1 network is activated in the RPT following an increase in intracellular Na^+^ (Na^+^_in_), resulting in an increase in Na,K-ATPase activity, in addition to the phosphorylation of Class IIa Histone Deacetylases (HDACs). In addition, activated SIKs repress transcriptional regulation mediated by the interaction between cAMP Regulatory Element Binding Protein (CREB) and CREB Regulated Transcriptional Coactivators (CRTCs). Through their transcriptional effects, members of the SIK family regulate a number of metabolic processes, including such cellular processes regulated during AKI as fatty acid metabolism and mitochondrial biogenesis. SIKs are involved in regulating a number of other cellular events which occur during AKI, including apoptosis, the Epithelial to Mesenchymal Transition (EMT), and cell division. Recently, the different SIK kinase isoforms have emerged as promising drug targets, more than 20 new SIK2 inhibitors and activators having been identified by MALDI-TOF screening assays. Their implementation in the future should prove to be important in such renal disease states as AKI.

## 1. Introduction

Salt Inducible Kinase (SIK) was first discovered in the adrenal gland of rats on a high salt diet, where it plays a regulatory role in steroidogenesis [1]. Subsequently, a SIK network was identified that plays an important role in regulating Na^+^ reabsorption in the Renal Proximal Tubule (RPT) [2]. SIK plays a number of additional roles, ranging from its roles in gene regulation, and the regulation of metabolism, to the roles played by SIK in cell survival, growth, the Epithelial to Mesenchymal Transition (EMT) as well as apoptosis. This report is concerned with the involvement of SIK in Acute Kidney Injury (AKI).

AKI is a heterogeneous group of conditions characterized by an abrupt decrease in the Glomerular Filtration Rate (GFR), followed by an increase in serum creatinine [3,4,5]. AKI arises as a consequence of ischemic and toxic insults, as well as radiation and ureteral obstruction [6,7]. Following the initiation and extension phases caused by the insults, the kidney goes through maintenance and recovery phases, during which repair occurs in cells that are sub-lethally damaged, in addition to the generation of new cells. There is a need to understand the underlying molecular changes that occur in the tubule epithelial cells during AKI, and the recovery period, so as to develop effective therapies. There is considerable evidence suggesting that signal transduction pathways that are activated during AKI involve different aspects of the SIK Networks, which opens up the possibility of new avenues for therapy.

## 2. Role of the Salt Inducible Kinase 1 (SIK1) Network in the Response of the Renal Proximal Tubule (RPT) to Injury

### 2.1. Initial Response of the Renal Proximal Tubule to Injury

Of particular interest in these regards, is the renal proximal tubule (RPT), one of the primary targets of injury in AKI [5]. The RPT primarily depends upon mitochondrial oxidative phosphorylation, rather than glycolysis [8]. Thus, ATP levels decline dramatically during hypoxia, resulting in a decrease in Na,K-ATPase activity, as well as an increase in intracellular Na^+^ (Na^+^_in_). As a consequence, the capacity of the RPT to reabsorb Na^+^ and other solutes is impaired. Impaired Na^+^ reabsorption can present problems, because in order to survive, the body must retain Na^+^ within an appropriate range (between 135 and 145 mEq/L). This level of Na^+^ is necessary to maintain a normal blood pressure, support the function of muscles and nerves, and to preserve our body’s fluid balance. One defense mechanism in the kidney, is Tubuloglomerular Feedback (TGF), which causes the Glomerular Filtration Rate (GFR) to decline when distal Na^+^ levels increase, and in this manner acts so as to preserve Na^+^ within a range compatible with renal tubular function (which may decline as a result of injury) [9]. In addition, tubule epithelial cells themselves possess defense mechanisms, including mechanisms that result in a response to changes in ionic balance. Although renal tubules are often viewed as being a central target in AKI, surprisingly little is known regarding these functional responses, which act to preserve renal tubule transport functions during AKI, including those in the RPT. Thus, it is important to gain an understanding of pertinent regulatory mechanisms involved in this nephron segment, and likely responses during AKI. Of particular interest in these regards is the SIK1 network, and its ability to regulate Na,K-ATPase, so as to maintain Na^+^ homeostasis in the body.

### 2.2. Importance of Na,K-ATPase in Na^+^ Reabsorption

As stated above, Na^+^ plays a critical role in maintaining the homeostasis of bodily fluids, indirectly affecting the distribution of water in intravascular compartments, and ultimately, blood pressure [10]. Fluid as well as electrolyte disorders often develop during AKI, and fluid therapy (with its own inherent problems) is common [11]. Indeed, clinically employed saline solutions (154 mM) contain higher Na^+^ levels than plasma (140 mM).

At the cellular level, the Na,K-ATPase is crucial in maintaining an appropriate Na^+^/K^+^ gradient across the plasma membrane (a requirement of all living cells). Presumably, the need for such a Na^+^/K^+^ gradient originated in primitive organisms, which were in an environment in which Na^+^ was inhibitory, or even toxic, while K^+^ was required for the activity of primitive enzymes (due to the preponderance of K^+^ in the primitive environment) [12]. Thus, cells had to develop mechanisms to prevent the toxicity of intracellular Na^+^. Indeed, if intracellular Na^+^ levels are allowed to rise in mammalian cells, the intracellular environment risks intracellular “flooding” caused by a Donan equilibrium (i.e., intracellular swelling caused by an increase in the intracellular concentration of free cations over and above the extracellular level, due to the net negative charge of intracellular proteins). For this reason, mammalian cells have a Na,K-ATPase which ejects three intracellular Na^+^ molecules in exchange for two extracellular K^+^, at the expense of ATP [13]. Very importantly, the Na^+^ gradient established by Na,K-ATPase provides the driving force for the cotransport of a number of solutes into the cell (amino acids, glucose, phosphate) with Na^+^. In the case of the RPT, this is the first step in the transepithelial transport of these solutes.

Teleologically, it makes sense that cells are fortified to survive the continual changes in the ions in the extracellular milieu, including those that occur during exposure to a “toxic” environment. Protection from such external changes is provided by signaling systems that control the activity of membrane transporters, including Na,K-ATPase, and thereby permit renal cells to respond appropriately to changes not only in the external ionic environment, but also to metabolic challenges such as a decline in ATP levels, as well as oxidative stress and other toxic challenges. Of particular interest in these regards is the SIK Signaling Network and its role in the RPT during AKI.

### 2.3. SIK Structure and Function

**Figure 1:**
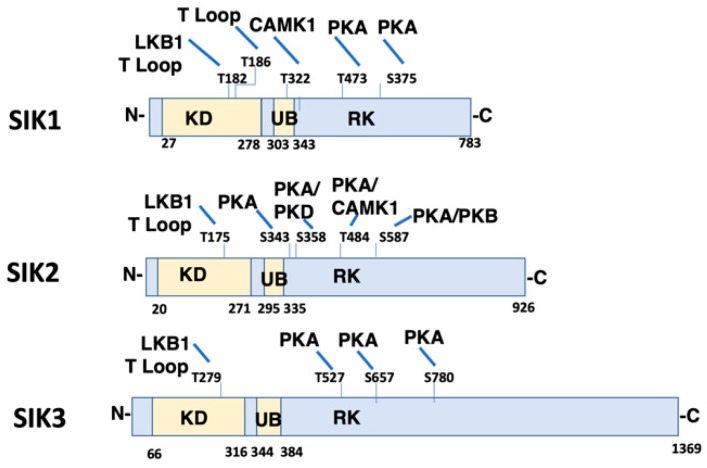
Domain Structure and Phosphorylation Sites of Salt Inducible Kinase (SIK) Isoforms. The domain structure of SIK1 (Uniprot P507059), SIK2 (Uniprot Q9Y2K2), and SIK3 (Uniprot Q9Y2K2) is shown including conserved Kinase Domain (KD), Ubiquitin Associated Domain (UB), and RK-rich region (RK). Identified phosphorylation sites are also illustrated, as well as the corresponding kinase (LKB1 (T182 SIK1, T175 SIK2, T221 SIK3), CaMK1 (T322 SIK1, T484 SIK2), PKA (T473 and S575 SIK1; S343, S358, T484 and S587 SIK2; T469, S551, S647 SIK3), and PKB (S587 SIK2)). T Loop indicates that the phosphorylation site is within the T activation loop. The amino and carboxy terminus of each SIK isoform are indicated by N- and C-, respectively. CaMK1, Calmodulin Activated Protein Kinase 1; PKA, Protein Kinase A; PKB, Protein Kinase B.

SIKs are central components of a number of signaling pathways which are responsible for regulating a wide range of cellular processes in different organs. In subsequent studies, SIK1 was found to be homologous to AMP Kinase (AMPK), and for this reason, is included within the Sucrose Non-Fermenting 1 (SNF1)/AMPK family [14]. Three isoforms of SIK have been identified (SIK1, SIK2 and SIK3) which share common structural features, including a highly conserved, amino terminal kinase domain, a Sucrose Nonfermenting-1 Homology (SNH) domain, as well as a carboxy terminal phosphorylation domain (Figure 1). All three SIK isoforms are broadly expressed in a large number of tissues. Tissues which express amongst the highest levels of SIK1 include adipose tissue, as well as B and T cells. While heart and adipose tissue are amongst those with the highest level of SIK2 expression, other tissues, including the cerebral cortex and testes are amongst those with the highest levels of SIK3. However, the relative SIK expression level is not a necessary indicator of the importance of an individual SIK isoform in any particular tissue. When considering the kidney, SIK1 has been studied most extensively, although the kidney expresses moderate levels of all three SIK isoforms.

SIKs possess an activation loop within the kinase domain. Autophosphorylation occurs within the activation loop (Thr186 in SIK1), an event that is essential for kinase activity [15]. The catalytic activity of all SIK isoforms depends upon the phosphorylation of yet another residue within the activation loop (Thr182 in SIK1) by LKB1 [16]. LKB1 similarly phosphorylates all other members of the AMPK family. The activation loop of SIK1 and SIK2 is also phosphorylated by GSK-3*β*, although this phosphorylation event is not sufficient to activate SIK kinase activity [15]. Other domains of the SIKs also undergo phosphorylation by a number of protein kinases. For example, Calmodulin Activated Protein Kinase 1 (CaMK1) phosphorylates SIK1 within the UB domain, so as to increase SIK1 kinase activity. In contrast, CaMK1 phosphorylates SIK2 within the carboxy terminal domain, and in this manner similarly activates SIK kinase activity. Protein Kinase A (PKA) phosphorylates all three SIK isoforms in the carboxy terminal domain, depending upon the physiological condition [17]. A consequence of SIK phosphorylation by PKA is that SIKs become associated with cytoplasmic 14-3-3 proteins, which results in the sequestration of SIKs in the cytoplasm [17]. Thus, SIKs that have been phosphorylated by PKA can no longer phosphorylate nuclear proteins, including CRTCs, and class IIa HDACs.

### 2.4. SIK1 Signaling Network and Na,K-ATPase

#### 2.4.1. Role of SIK1 in Na^+^ Sensing: Acute Response

**Figure 2:**
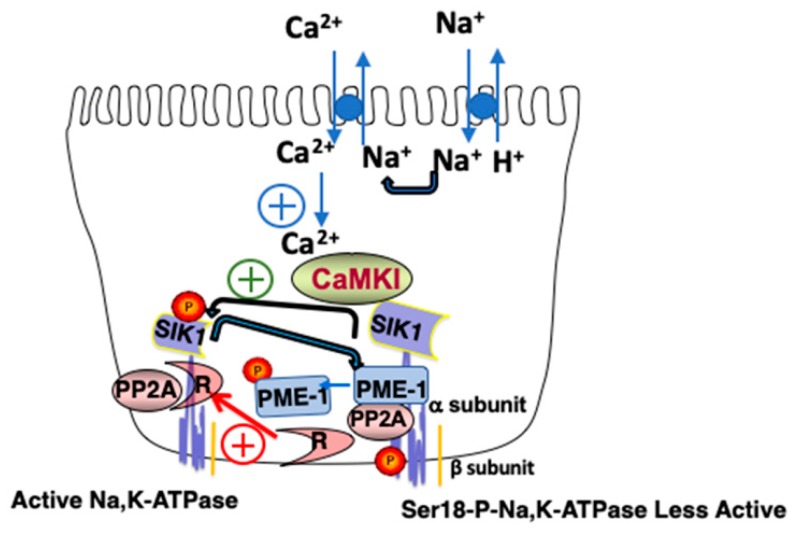
Model of the Basolateral SIK1 Network. An increase in luminal Na^+^ results in an increase in Na^+^_in_, the activation of Na^+^/Ca^2+^ exchange, and an increase in Ca^2+^_in_, which activates CaMK1. Activated CaMK1 phosphorylates SIK1 at Thr322 and activates basolateral SIK1, which as a consequence, phosphorylates PME-1, which dissociates from the Na,K-ATPase complex. PP2A remains demethylated, associates with its regulatory subunit (R), and in this activated state, dephosphorylates Ser18 of the catalytic subunit of Na,K-ATPase (i.e., the *α* subunit), activating the transporter. If the indicated reaction has a positive effect upon activity, it is indicated by a plus contained within a circle.

Of particular interest to this report, is the role of SIK1 in Na^+^ sensing. In RPT cells, SIK1 is associated with a complex associated with the basolateral membrane, containing the Na, K-ATPase [12]. A number of proteins are associated with the SIK1/Na,K-ATPase complex, including Protein Phosphatase 2A (PP2A), and Protein Methylesterase-1 (PME-1), which constantly demethylates (and thus inactivates) PP2A. As illustrated in Figure 2, the increase in intracellular Na^+^ (Na^+^_in_), which occurs following an increase in luminal Na^+^, is followed by the activation of Na^+^/Ca^2+^ exchange activity, and, as a consequence, an increase in intracellular Ca^2+^ (Ca^2+^_in_).

Ultimately, the increase in Ca^2+^_in_ results in CaMK1 activation, which in turn phosphorylates and activates SIK1. SIK1 then activates basolateral Na,K-ATPase, albeit indirectly [12]. Towards these ends, activated SIK1 phosphorylates PME1, which dissociates from the SIK1/Na,K-ATPase/PP2A complex. As a consequence, the PP2A catalytic subunit is no longer demethylated by PME1, and thus the activity of PP2A remains low. This is because PP2A only achieves its full catalytic activity following its demethylation, which allows the PP2A catalytic subunit to interact with the PP2A regulatory subunit, and dephosphorylate the catalytic subunit (i.e., the *α* subunit) of the Na,K-ATPase. The retention of the Na,K-ATPase in the basolateral membrane increases under these conditions, resulting in increased transport activity.

#### 2.4.2. Relevance of the SIK1 Signaling Network and Na,K-ATPase to AKI

The SIK1 signaling network described above is responsible for eliciting the initial responses of the RPT to insults that result in AKI. An increase in luminal Na^+^ (Na^+^_in_) occurs in response to a number of these insults, particularly when the GFR decreases (during the initiation phase of AKI), and luminal Na^+^ increases. Intracellular ATP levels often decline during the initiation phase of AKI (often in response to hypoxia). A consequence of such a decline in intracellular ATP is reduced Na,K-ATPase activity, and, as a result, an increase in Na^+^_in_ (which decreases the driving force for transepithelial transport). From our knowledge of the SIK1 network [12], such acute increases in Na^+^_in_ rapidly activate the basolateral SIK1s, which are part of the Na,K-ATPase complex, and thereby initiate the events in the SIK1 network which counteract the tendency for Na^+^ reabsorption to decline during AKI (by increasing the quantity of basolateral Na,K-ATPases).

In addition to these immediate effects of an increase in Na^+^_in_ (which occurs during the initiation phase of AKI), during subsequent phases of AKI changes in gene expression occur which result in increased renal reabsorption, as well as other repair processes, due to changes occurring in other aspects of SIK networking.

### 2.5. Transcriptional Effects of SIK1 Involving Its HDAC Kinase Activity and Relevance to AKI

**Figure 3:**
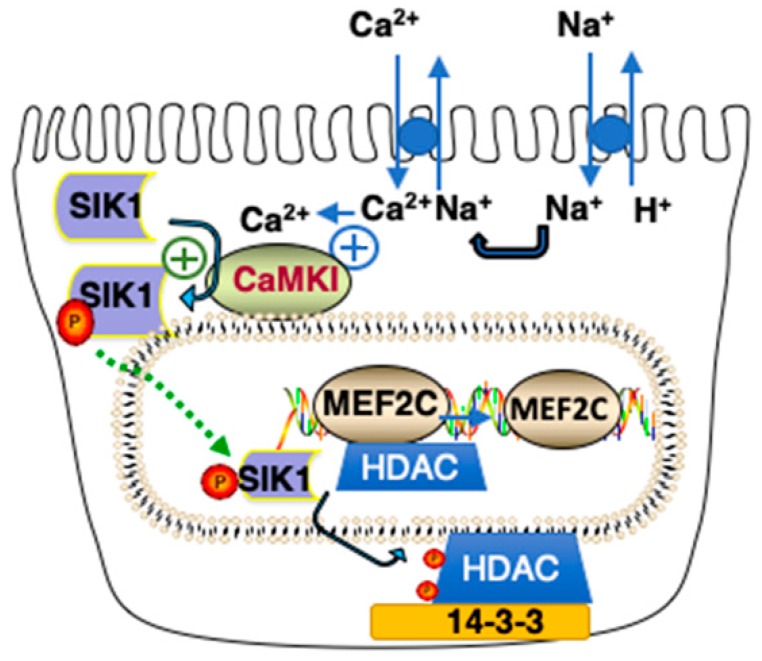
Model of Class IIa HDAC Kinase Phosphorylation by SIK1. An increase in Na^+^_in_ results in an increase in Ca^2+^_in_ due to activation of Na^+^/Ca^2+^ exchange activity. SIK1 is phosphorylated at Thr322 by CAMK1 and activated, resulting in the phosphorylation of Class IIa HDACs associated with MEF2 transcriptions (as well as NFAT, not shown) on chromosomal DNA. As a consequence of their phosphorylation at two sites (Ser259 and Ser498 in the case of Human HDAC5), Class IIa HDACS translocate to the cytoplasm where they interact with 14-3-3 proteins. If the indicated reaction has a positive effect upon activity, it is indicated by a plus contained within a circle.

During the initiation phase of AKI, Na^+^_in_ increases, resulting in SIK1 activation, which in turn results in a transcriptional response due to the Class IIa Histone Deacetylase (HDAC) kinase activity of SIK1 [18,19]. Class IIa HDACs associate with such transcription factors as Myocyte Enhancer Factor 2C (MEF2C) and Nuclear Factor of Activated T cells (NFAT) on chromosomal DNA, so as to repress transcription (Figure 3). However, when SIK1 is phosphorylated at Thr322 by CAMK1 (and activated), activated SIK1 phosphorylates Class IIa HDACs, which, as a consequence, a) dissociate from chromosomal MEF2 (as well as NFAT), and b) translocate to the cytoplasm, where they bind to cytoplasmic 14-3-3 proteins (Figure 3).

Following the dissociation of Class IIa HDACs from chromosomal DNA, transcription via MEF2 and NFAT is upregulated, increasing the expression of MEF2- (and NFAT-) regulated genes. Included amongst the genes upregulated following an increase in Na^+^_in_ are those encoding for Atrial Natriuretic Peptide (ANP) as well as *α* and *β* Myosin Heavy Chains (MHCs) [18]. Of particular interest in these regards, is the increased expression of ANP A and B, as well as *α* and *β* MHCs which has been reported during AKI [20,21,22]. Such an increase in the expression of ANP, may very well promote recovery during AKI. Indeed, ANP has been used in the management of AKI [23,24,25].

### 2.6. Involvement of SIK1 in Transcriptional Events Mediated by cAMP and Ca^2+^

SIKs are also involved in regulating transcriptional events mediated by the cAMP Regulatory Element Binding Protein (CREB) and CREB Regulated Transcriptional Coactivators (CRTCs) [26]. Prostaglandin E_2_ (PGE_2_) is an example of an effector that acts via such a mechanism [27,28]. In the kidney, PGs are synthesized from Arachidonic acid by Cyclooxygenases (COXs), including constitutive COX1 and inducible COX2. Of particular interest in these regards, COX-2 inhibitors are a major cause of drug-induced AKI, and exacerbate reductions in the GFR caused by other agents such as Lipopolysaccharides (LPS) [29]. In addition, chemokines and cytokines including PGE_2_ are produced during AKI (as early as the initiation phase) [9,29]. While they may have beneficial effects on the GFR, chemokines and cytokines also contribute to the inflammatory response [29].

PGE_2_ in particular is known to regulate renal Na^+^ handling, in a manner which depends upon the nephron segment [9]. PGE_2_ interacts with its specific G Protein Coupled Receptors (GPCRs) (i.e., Prostaglandin E (EP) receptors) which are differentially expressed in tubule epithelial cells. Included amongst the GPCRs for PGE_2_ are EP2 and EP4 receptors (which are coupled to Gs, which activates adenylate cyclase (AC)), as well as EP1 receptors (which is coupled to Gq, which activates Phospholipase C (PLC)) [30]. Following the interaction of PGE_2_ with Gs coupled EP2 and EP4 receptors, intracellular cAMP increases, resulting in the phosphorylation SIK1 at Thr475 (by PKA), and SIK1 inactivation [27,31,32], unlike increases in Na^+^_in_ which result in SIK1 phosphorylation at Thr322 (by CAMK1), and SIK activation.

The PGE_2_-mediated increase in Na,K-ATPase activity in primary rabbit RPT cell cultures is an example of transcriptional regulation which involves inhibitory effects of cAMP on SIK1 [33]. The Na,K-ATPase is a heterodimer, consisting of an *α* subunit, responsible for the catalytic activity, as well as a *β* subunit, a chaperone which facilitates the insertion of the Na,K-ATPase into the basolateral membrane. Although both subunits are present in Na,K-ATPase, an increase in the level of the *β* subunit alone can cause an overall increase in the level the heterodimer [34]. This occurs when the level of the *β* subunit is limiting to heterodimer formation (excess *α* subunit being degraded). In this case, transcriptional regulation of the Na,K-ATPase *β* subunit gene *atp1b1* becomes a critical determinant of the overall Na,K-ATPase level, and activity. The observed increase in the overall level of the Na,K-ATPase in the RPT cells following a prolonged incubation with PGE_2,_ can be explained by this type of regulation by the *atp1b1* gene [35].

**Figure 4:**
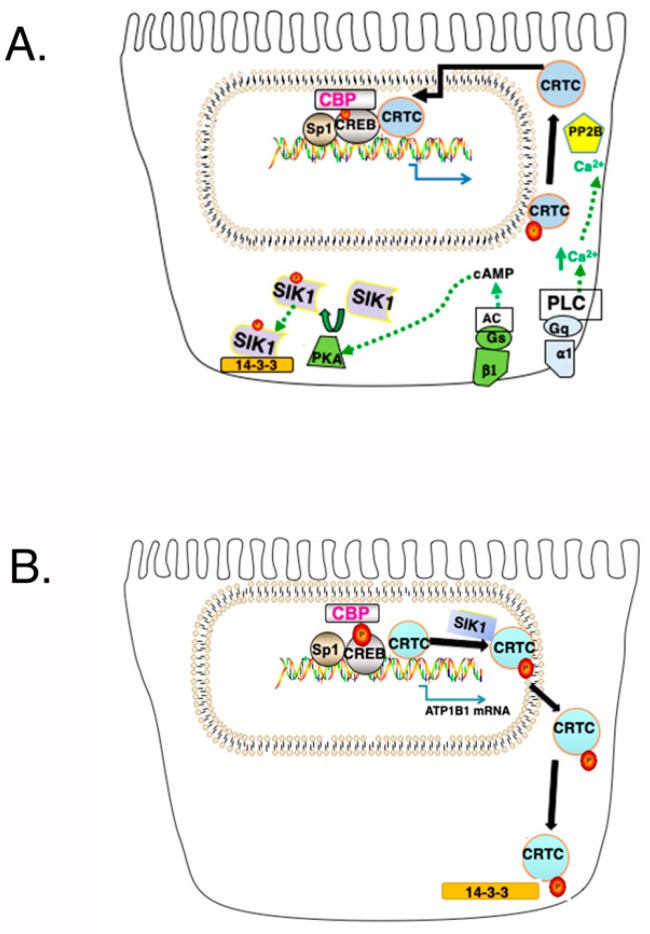
Establishment of a CREB/CRTC Interaction and its Disruption by SIK1. (**A**) Following a simultaneous increase in cAMP and Ca^2+^_in_, SIK1 is phosphorylated at Thr473 by PKA, which results in an interaction between PKA phosphorylated SIK1 and 14-3-3 proteins (which results in the sequestration of SIK1 in the cytoplasm, and which prevents the further phosphorylation of CRTCs by SIK1). CRTCs, which are phosphorylated (at Ser151 in the case of human CRTC1), are dephosphorylated by Calcineurin (i.e., Protein Phosphatase 2B (PP2B)). As a consequence, CRTCs translocate to the nucleus and interact with CREB, causing an increase in transcription, beyond that obtained with the Ser-133-pCREB/CBP interaction. (**B**) Following SIK1 activation (and its dephosphorylation at the PKA phosphorylation site), SIK1 no longer interacts with 14-3-3 proteins, which permits the nuclear translocation of SIK1. Once in the nucleus, SIK1 phosphorylates CRTCs, which translocate to the cytoplasm.

Transcriptional regulation of the *atp1b1* gene by PGE_2_ involves CREB, which binds to Prostaglandin Regulatory Elements, PGREs, located in the *atp1b1* promoter [36,37]. CREB binds constitutively to PGREs, even in the absence of the binding of effectors such as PGE_2_ to their receptors (i.e., EP receptors in the case of PGE_2_) (Figure 4A). Under these conditions, CREB Regulated Transcriptional Coactivators (CRTCs) are localized in the cytoplasm. Transcriptional regulation by CREB on the *atp1b1* promoter involves increases in cAMP as well as Ca^2+^_in_. PGE_2_ interacts with both EP2, as well as EP1 receptors in primary RPT cells. When PGE_2_ interacts with EP2 receptors, AC is activated, and subsequently, PKA. Activated PKA phosphorylates CREB (localized on the PGRE1 and PGRE3 sites on the *atp1b1* promoter), resulting in the recruitment of CREB Binding Protein (CBP) to phospho-CREB (pCREB) [36]. In order to obtain a maximal transcriptional response, CREB Regulated Transcriptional Coactivators (CRTCs) also associate with CREB on the *atp1b1* promoter [27] (Figure 4B). However, a second signaling event (an increase in Ca^2+^) must occur if CRTCs are to interact with CREB [27,38]. This second signaling event is initiated by the interaction of PGE_2_ with EP1 receptors, the activation of Phospholipase C (PLC), and an increase in Ca^2+^_in_ [31].

Notably, the binding of CRTCs to CREB on the *atp1b1* promoter is limited by SIK1 [27]. In order for CRTCs to interact with CREB, CRTCs must be in the nucleus (Figure 4A). However, activated SIK1 phosphorylates CRTCs, which causes CRTCs to translocate to the cytoplasm where they interact with 14-3-3 proteins (Figure 4B) [27].

Two simultaneous signaling events must occur in order for cytoplasmic CRTCs to reenter the nucleus (Figure 4A) [39]. First, intracellular cAMP must increase (due to the activation of AC, by Gs coupled EP2 in the case of PGE_2_), and second, Ca^2+^_in_ must increase (due to the activation of PLC, by Gq coupled EP1 in the case of PGE_2_). Following an AC-mediated increase in cAMP, PKA (which is activated) phosphorylates SIK1 at Thr475, inactivating SIK1, which no longer phosphorylates CRTCs. CRTCs must in addition be dephosphorylated in order to enter the nucleus. The dephosphorylation of CRTCs depends upon the activation of Calcineurin, which occurs when Ca^2+^_in_ increases. Calcineurin (i.e., Protein Phosphatase 2B (PP2B)), is a calcium- and calmodulin-dependent serine/threonine protein phosphatase.

### 2.7. Summary of the Three Major SIK1 Networks and Their Relevance to Acute Kidney Injury (AKI)

Thus, to summarize, the regulation of the transport and reabsorptive functions of the RPT depends upon three different signaling pathways involving SIK1. Two of these pathways are activated when Na^+^_in_ increases (which occurs following increases in luminal Na^+^), and involve on one hand, the direct phosphorylation of PME1 by activated SIK1 basolateral SIK1 (in the first pathway), and on the other hand the direct phosphorylation of class IIA HDACs by activated nuclear SIK1 (in the second pathway). As a consequence, 1) pre-existing Na,K-ATPases are retained in the basolateral membrane in the first pathway, and 2) transcriptional regulation by MEF2 and NFAT transcription factors increases in the second pathway. A third component of the SIK1 network, transcriptional regulation by CRTCs (and CREB), is inhibited when SIK1 is activated, due to the phosphorylation of CRTCs by SIK1. This latter event may occur in the absence of an increase in Na^+^_in_ (i.e., when SIK1 is activated by LKB1 or CAMK).

The two SIK1 signal transduction pathways, which are activated following an increase in Na^+^_in_, both play a role during the initial phase of AKI. During this phase, a) luminal Na^+^ increases due to TGF, and b) intracellular ATP levels may decline due to ischemia and/or a toxic insult. In this latter case, Na^+^ extrusion by Na,K-ATPase declines, as this transporter depends upon cellular energy levels. As a consequence, Na^+^_in_ increases further, resulting in a) a further activation of SIK1, and increase in Na,K-ATPase activity (via PP2A), as well as b) protective effects elicited as a consequence of the expression of NFAT-regulated genes (a result of the class IIa HDAC Kinase activity of SIK1). During these responses, activated SIK1 can also restrict chronic, transcriptional regulation of Na,K-ATPase by CREB (through the phosphorylation of CRTCs).

The restrictive effect of SIK1 on transcriptional regulation of Na,K-ATPase by CREB and CRTCs is alleviated, however, when SIK1 is phosphorylated, and inhibited by PKA. This occurs in response to cytokines (such as PGE_2_) which are produced during the maintenance and recovery phases of AKI. During this time, the necessary increases in intracellular cAMP and Ca^2+^_in_ required for the interaction of CRTCs with CREB on the *atp1b1* promoter must occur if RPT transport activity is to increase over the long term.

The SIK1 signaling networks described above are evolutionarily highly conserved, including the SIK1 signaling events responsible for maintaining cellular Na^+^ homeostasis [40]. SIK1 orthologues have been reported in a diverse range of animal cells ranging from mouse, zebrafish, and drosophila, to Caenorhabditis elegans [12]. Even plants express the orthologous SOS2 and SOS3, which permit adaptions to drought, as well as increases in the salinity of the soil [41].

## 3. Other Roles of SIKs

### 3.1. Roles of SIK2 and SIK3 in Gluconeogenesis and Lipogenesis

SIKs are regulators of a diverse range of cellular functions which are much broader than those activated by ionic imbalances. Indeed, each of the three different SIK isoforms plays a variety of roles, in different tissues [26]. Examples discussed below include the role of SIK3 in hepatic gluconeogenesis [42], as well as the role of SIK2 in lipogenesis in adipocytes [43].

#### 3.1.1. Role of SIK2 and SIK3 in Gluconeogenesis in the Liver

When blood glucose levels are high, insulin, which is produced, stimulates the phosphorylation and activation of Akt in the liver [38]. Akt in turn phosphorylates hepatic FOXO transcription factors, which become sequestered in the cytoplasm. Under these conditions, SIK3, which is activated by LKB1, phosphorylates CRTC2 and CRTC3 (as well as Class IIa HDACs), resulting in the sequestration of these proteins in the cytoplasm. As a consequence, gluconeogenic gene expression is repressed.

In contrast, during fasting, glucagon is produced, with a resultant increase in hepatocyte cAMP, the activation of AC, which as a consequence, phosphorylates (and inactivates) SIK3 [38]. Subsequently, CRTC2 and CRTC3 are dephosphorylated by calcineurin, and translocate to the nucleus, where they interact with pCREB (and CBP) on the promoters of gluconeogenic genes, including genes encoding for Phosphoenol Pyruvate Carboxykinase (PEPCK) and Glucose 6 Phosphatase (G6Pase).

At the same time (i.e., during fasting), hepatic class IIa HDACs and FOXO1 become dephosphorylated (due to the inhibition of SIK3), and translocate to the nucleus [44]. HDAC4 and HDAC5 (Class IIa HDACs) interact with FOXO1 on IRE sites in the PEPCK and G6Pase promoters, where they recruit HDAC3 (a Class I HDAC), which deacetylates FOXO1, thereby stimulating transcription. Thus, both CRTCs and Class IIa HDACs act so as to stimulate the expression of these hepatic gluconeogenic genes.

#### 3.1.2. Role of SIK2 and Activating Transcription Factor 3 (ATF3) in Lipid Metabolism in Adipocytes

SIKs, HDACs and CRTCs similarly play a role in transcriptional regulation in other insulin-sensitive tissues, including adipocytes [38]. In white adipocytes, catecholamines (which are produced by the hypothalamus during fasting) stimulate lipolysis by activating *β* adrenergic receptors (and thus PKA) resulting in the phosphorylation of hormone-sensitive lipase [45]. Activated PKA also phosphorylates, and inactivates SIK2, which results in an increase in the transcription of ATF3 (ATF3 being a CREB, and CRTC dependent gene). ATF3 acts as a transcriptional repressor of the insulin responsive GLUT4 gene, as well as the adiponectin gene. In contrast, in the fed state SIK2 becomes dephosphorylated and activated, which results in the phosphorylation of CRTC2 (as well as HDAC4), and reduced expression of ATF3. The ultimate result is increased expression of GLUT4 and glucose uptake, promoting lipogenesis.

#### 3.1.3. Relevance to the Kidney

Although the kidney is not generally noted as being an insulin-sensitive tissue, the RPT nevertheless does have gluconeogenic capacity, that is reportedly inhibited by insulin as well as glucose, by a mechanism which involves the inactivation of FOXO1 [46]. Very likely this mechanism involves SIK3, which very likely phosphorylates class IIa HDACs in the kidney (as in the liver), thereby preventing the interaction of class IIa HDACs (and HDAC3, a Class I HDAC) with FOXO1 on IREs in the PEPCK and G6Pase promoters (an interaction which is very likely just as necessary for the activation of FOXO1 in the kidney, in addition to the liver). Little is known about the effects of catecholamines on ATF3 expression in the kidney. However, ATF3 is rapidly induced during ischemia/reperfusion injury, and suppresses the expression of inflammatory cytokines (including IL-6 and IL-12b) during AKI (as indicated by studies with AFT3 deficient mice) [47]. Catecholamines (or other renal effectors that activate PKA) may be responsible for the induction of ATF3 in the kidney during AKI by stimulating the phosphorylation and inactivation of SIK2 by PKA (as observed in white adipocytes), thereby resulting in increased transcription of AFT3 (due to a CREB/CRTC1 interaction in the kidney as occurs in white adipocytes).

### 3.2. Role of SIK2 in Mitochondrial Biogenesis and Its Relevance to AKI

During AKI, the ability of the RPT to carry out is reabsorptive functions is impaired in part due to a reduced capacity to generate sufficient levels of ATP. In addition, Reactive Oxygen Species (ROS) are generated due to injuries affecting the mitochondria. These problems can be alleviated by means of mitochondrial biogenesis, which depends upon the expression of the Peroxisome Proliferator-Activated Receptor *γ* Co-activator-1*α* (PGC-1*α*) gene, which in turn depends upon SIKs.

#### 3.2.1. Role of SIK2 in Regulating PGC-1*α* Adipocytes

In adipocytes, the PKA mediated activation of CREB, and its interaction with CRTC2 (which occurs following the inhibition of SIK2), results in increased expression of PGC-1*α* [48]. PGC-1*α* functions to induce Uncoupling protein 1 (UCP-1), as well as other mitochondrial genes and transcription factors. The ultimate result is mitochondrial biogenesis, and an increased oxidative capacity. In white adipocytes, inducers of PGC-1α include *β* adrenergic agonists, thyroid hormone, and in brown adipocytes, insulin. Insulin acts as an inducer in brown adipocytes by activating Phosphoinositol 3-Kinase (PI3K) as well as Akt, and ultimately phosphorylating SIK2 (at Ser587). In contrast, SIK1 has been implicated in regulating the expression of PGC-1*α* in skeletal muscle [49]. In skeletal muscle, important promoter elements for the PGC-1*α* gene include a regulatory element for CREB (i.e., a CRE), as well as MEF2. Thus, SIK1 regulates PGC-1*α* gene expression via its ability to phosphorylate CRTCs [50], as well as class IIA HDACs [19].

#### 3.2.2. Regulation of Mitochondrial Biogenesis during AKI

In the RPT, mitochondrial biogenesis is particularly important in maintaining the energy demands of the RPT after injury, including injuries that result in AKI [51]. This is because the RPT primarily depends upon mitochondrial oxidative metabolism to produce the ATP necessary to carry out its reabsorptive functions, having a very limited capacity for glycolysis [29]. Thus, following injuries to the RPT, the remaining viable, transporting RPT cells may very well require additional mitochondria in order to meet their energy requirements.

Several small molecules such as resveratrol and isoflavone-derived compounds, induce mitochondrial biogenesis in the RPT, and have been used to alleviate deleterious effects of injuries to the RPT [52]. While resveratrol has been observed to increase mitochondrial biogenesis through the activation of AMPK in fibroblasts, a similar AMPK activation has not been observed in RPT cells [52]. Thus, the SIK network may be involved. Indeed, a recent report indicates that the mitochondrial biogenesis that occurs after the formoterol-induced recovery from ischemia-reperfusion injury is mediated by *β* adrenergic receptors [53]. Similarly, mitochondrial biogenesis occurs in brown adipocytes in response to catecholamines and occurs via a mechanism which involves the phosphorylation and inhibition of SIK2 [48].

The induction of mitochondrial biogenesis following injury to the RPT is also an important means of overcoming damage caused by Reactive Oxygen Species (ROS). ROS production occurs more often when the mitochondrial electron transport chain becomes more reduced as a consequence of cellular injuries [4,54,55]. This problem is alleviated when mitochondrial biogenesis occurs, which causes oxidative substrates to be distributed to more mitochondria. As a consequence, the degree to which the electron transport chain becomes reduced in individual mitochondria (and hence the degree that ROS production occurs) decreases.

As stated above, mitochondrial biogenesis is mediated by PGC-1*α*, whose expression depends upon the interaction of CRTCs with CREB, which in turn depends upon SIK. Following its induction, PGC-1*α* interacts with FOXO3 so as to increase the expression of the antioxidant genes CAT, SOD2, and GPX1 (which encode for catalase, superoxide dismutase and glutathione peroxidase, respectively) [55,56]. Indeed, these same antioxidant enzymes are induced in the kidney response to AKI [57,58]. Therefore, an understanding of the role of the SIK network in this process is important.

### 3.3. Yet Other Roles Known to Be Played by SIKs in Events Which Occur during AKI

A number of other metabolic events and cellular processes that occur during AKI are also known to involve SIKs. Included amongst the metabolic events is the decline in Fatty Acid (FA) oxidation in the RPT, which results in the Epithelial to Mesenchymal Transition (EMT). In addition, lethal damage during AKI results in apoptosis, and sublethal damage in repair processes, and ultimately cell division. The decline in FA oxidation, and apoptosis, as well as the progression of cells through the cell cycle, and ultimately, cell division involve SIKs.

#### 3.3.1. Fatty Acid Oxidation

##### ATP Production in the RPT Is Generated by FA Oxidation, Which Declines in AKI

As stated above, RPT cells are included amongst the most energy demanding cells in the body, expending extensive quantities of ATP in order to meet their reabsorptive requirements. However, following a hypoxic or toxic insult, there is a major decline in cellular ATP and metabolism. ATP production by the RPT is primarily generated by the oxidation of FAs in mitochondria as well as peroxisomes. This process is largely shut down during AKI and for a lengthy period thereafter [54]. The FAs utilized by RPT cells are generated by their uptake by FA transporters such as CD36, the deacylation of phospholipids, and other metabolic events. Free FAs are first metabolized by Carnitine Palmitoyl-Transferase 1 (CPT-1), so as to form acyl carnitine derivatives, which enter mitochondria and peroxisomes [54]. After entering mitochondria and peroxisomes, the metabolism of FAs then depends upon enzymes including mitochondrial medium chain acyl COA dehydrogenase (MCAD) and peroxisomal acyl-CoA oxidase (ACOX1). However, both CPT-1 and ACOX1 enzymatic activity declines following ischemia/reperfusion injury, and MCAD activity declines as a consequence of cisplatin-induced AKI [54]. RPT cells have only a limited capacity to metabolize glucose in lieu of FAs. Thus, the decline in ATP levels that occurs in the RPT during AKI can be prolonged, with severe consequences.

##### SIK2 Regulates FA Oxidation in Other Tissues

Of particular interest in these regards, are the studies indicating that SIK2 regulates FA oxidation, in the liver, skeletal muscle, as well as in adipocytes [45]. Indeed, the levels of ACOX1, CPT1 and MCAD all decline in SIK2 KO mice. Thus, inactivation of SIK2 is an explanation for the decline in FA oxidation in RPT cells during AKI. Presumably then, interventions which specifically result in SIK2 activation would be expected to counteract the decline in FA oxidation (and resulting reductions in ATP levels).

##### Decreased FA Oxidation Results in EMT in the RPT

A number of studies indicate that the decrease in FA Oxidation that occurs during AKI results in EMT [54]. For example, studies with HK2 cells indicate that such a decrease in FA oxidation, and lipid accumulation precedes a glucose-induced morphological change typical of the EMT. Similarly, RPT cell cultures treated with the CPT-1 inhibitor Etoxmoxir undergo EMT. However, an alternative explanation for the EMT observed in the RPT during AKI (as well as during a decline in FA oxidation) is that it is a result of a TGF*β*-mediated downregulation of cell–cell junctional constituents including E-cadherin.

##### Involvement of SIK1 in EMT

The studies of Vanlandewijck et al. [59] indicate that TGF*β* downregulates E-cadherin through the activation of SIK1, which phosphorylates the polarity complex protein Par3 (a regulator of tight junction assembly), resulting in the degradation of Par3 by the proteasomes and lysosomes, an event which ultimately results in EMT. In addition, other kinases such as LKB1 maintain epithelial cell polarity, by activating SIKs, causing transcriptional repressors such as Snail1 to be downregulated (thereby preventing repression of E-Cadherin) [60].

TGF*β* is a key profibrotic factor that is activated during AKI, and promotes EMT in the RPT [61]. Following repeated injury, RPT cells which have undergone EMT may fail to either re-differentiate, or to regain normal mitochondrial and metabolic function. Instead, these damaged RPT cells produce large quantities of TGF*β*, which perpetuates the EMT, and prevents the proliferation necessary for repair. As a consequence, events can be initiated which result in Chronic Kidney Disease (CKD) [61]. Presumably, TGF*β* production is responsible for perpetuating these problems. Conceivably, this cycle of events may be prevented by the use of SIK1 inhibitors to prevent signal transduction events which have been initiated via TGFβ receptors.

#### 3.3.2. Role of SIKs in Apoptosis

Transient ischemia which occurs as a result of hypovolemia, hypotension and/or heart failure is a common cause of AKI. As stated above, the RPT is particularly susceptible to ischemic damage, which results in their detachment from their substratum, and death (either by apoptosis or necrosis) [62]. The apoptosis which occurs in the RPT during ischemia is a means of removing damaged, dysfunctional cells from the kidney. A number of studies indicate that p53 plays an important role in the apoptosis, cell cycle arrest and autophagy that occurs in the RPT when AKI is induced by ischemia, cisplatin and even folic acid [62]. Of particular interest in these regards are recent studies indicating that p53-dependent anoikis (a subtype of apoptosis resulting from a lack of cell adhesion) is mediated by SIK1 [63].

#### 3.3.3. Pro-Survival Roles of SIKs and Epigenetics

##### Prosurvival Role of SIK1 in Myocytes Involves SIK1’s HDAC Kinase Activity

A number of studies indicate that in addition, SIK1 has a pro-survival role (pertinent to cells which have been damaged, but are not destined to undergo apoptosis). Studies with transgenic mice with the targeted expression of a dominant negative CREB (ACREB), indicated that the survival of the differentiated cells in the targeted tissues decreased in the absence of functional CREB protein [19,64,65,66]. Initially, investigators thought that CREB was directly responsible for increasing cell survival and differentiation in the targeted tissues. However, subsequent studies indicated that CREB promoted cell survival (and differentiation) indirectly, by inducing the expression of the Snf1lk gene (encoding SIK1), resulting in an elevated level of activated SIK1 [19]. The activated SIK1 was observed to phosphorylate Class IIa HDACs, an event which was lost in vitro when the SIK1 phosphorylation site was removed by mutation [67]. The ultimate result of SIK1 phosphorylation in vivo was increased cell survival.

The initial studies indicating that SIK1 promoted cell survival were conducted with mice in which ACREB was targeted to skeletal muscle [67]. In skeletal muscle, previous studies indicated that the myogenic program depends upon MEF2 transcriptional activity. The binding of Class IIa HDACs to MEF2 transcription factors on chromosomal DNA inhibits MEF2-mediated transcription, and thus myogenesis. However, CREB activation was observed to induce SIK1 expression (which no longer occurred in ACREB skeletal muscle). Normally, the phosphorylation of Class IIa HDACs by SIK1 (and their translocation to the cytoplasm), results in increased MEF2 transcriptional activity and myogenesis (events which are impaired in ACREB mice). The discovery of that SIK1 is a pro-survival factor that promotes myogenesis, resulting in a reassessment of many previous studies.

##### Involvement of Epigenetics and Class IIa HDACs in Regeneration of Damaged Kidneys

A number of recent studies indicate that a number of epigenetic events, including histone acetylation, are important in the regeneration of damaged kidneys [68]. Indeed, a decrease in histone acetylation has been reported in RPTs subjected to energy depletion, while during the subsequent recovery period HDAC5 (a class IIa HDAC) was downregulated, and histone acetylation increased [69]. SIK1 may very well play a role in this process.

#### 3.3.4. Role of SIKs in Cell Growth and Hypertrophy

##### Cell Division and Hypertrophy during AKI

In the normal kidney, RPT cells divide very slowly [70]. Cell division only occurs as a means to replace tubule epithelial cells, which are lost very slowly into the urine. However, following an ischemic or toxic insult, many RPT cells, which were previously quiescent, enter the cell cycle (even when there is massive necrosis and apoptosis). Many of the tubule epithelial cells which enter the cell cycle immediately after an ischemic or toxic insult spend a lengthy period in G2/M, secreting factors such as TGF*β*, which result in fibrosis [71]. A large proportion of the epithelial cells which enter the cell cycle immediately after an ischemic or toxic insult have extensive DNA damage, leading to a mitotic catastrophe [70].

Subsequently, during the recovery period of AKI, additional RPT cells, including those which are sub-lethally injured, enter the cell cycle, and may divide [29]. The newly generated RPT cells migrate to regions which have been denuded as a consequence of injury, where they assume the polarized morphology required for transepithelial transport.

In recent studies, Lazzeri et al. [72] tracked the fate of individual tubular cells in conditional Pax8/Confetti mice during AKI. The results indicate that regeneration of new tubule epithelial cells during AKI is not as extensive as previously thought, even though kidney function recovers. Renal functional recovery could be attributed to some of the original tubule epithelial cells, in addition to limited progenitor driven regeneration. A number of the original tubule epithelial cells (or remnant cells) which were in the cell cycle, actually, went through endocycles (i.e., alternative cell cycles without cell division). Tubule epithelial cells which had undergone endoreplication cycles were often polyploid, and had undergone hypertrophy. Notably, endocycling cells and hypertrophy was also observed in the kidneys of patients after AKI.

##### Role of SIKs in Hypertrophy and Cell Division

As discussed above, the hypertrophy of atrial myocytes has been attributed to the activation of SIK1, in particular following an increase in intracellular Na^+^ [18]. Recent studies by Popov et al. [73] indicate that SIK2 activation also causes hypertrophy in cardiac myocytes. In rats expressing a hypertensive variant of the *α*-adducin gene, the expression of SIK2 is elevated, as well as genes associated with left ventricle hypertrophy (LVH). Similarly, in mice on a high salt diet, LVH can be prevented by the ablation of sik2. Although not reported in the case of cardiac tissue, the SIK3 isoform is essential for chondrocyte hypertrophy during skeletal development [74]. However, the SIK Isoform(s) involved in renal hypertrophy have not been identified.

A number of recent studies indicate that SIKs regulate cell proliferation, particularly under conditions of stress. SIK2 promotes G1/S progression presumably due to its ability to phosphorylate the p85 *α* subunit of Phosphoinositide 3 Kinase (PI3K) [75], which mediates signaling in response to a number of growth factors. In addition, SIK2 is localized in centrosomes, where it acts as a “centrosome kinase” required for mitotic spindle formation [76]. The SIK3 isoform similarly is a mitotic regulator [77,78]. Indeed, mitosis is extended following a SIK3 knockdown. The increased duration of mitosis following a SIK3 knockdown has been attributed to a SIK3 requirement for mitotic exit. Although the metaphase plate forms normally when SIK3 is depleted, the onset of anaphase is delayed.

## 4. Summary and Therapeutic Potential

All three SIK isoforms are expressed in the RPT, and most likely play distinct roles in the preservation and reacquisition of renal function during AKI. Presently, the role played by each of the SIK isoforms during AKI can be surmised from investigations conducted with the renal SIK1 network, as well as with the SIK2 and SIK3 isoforms in other tissues, as summarized below.

During the initiation and extension phases of AKI, renal reabsorptive function declines and cytokines are produced. Activation of the SIK1 network during this time period results in an acute increase in the number of basolateral Na,K-ATPases (due to a SIK1 mediated decrease in basolateral PME activity, and an increase in dephosphorylated Na,K-ATPase (by activated PP2A)). The SIK1 activation caused by an increase in Ca^2+^_in_, also results in an increase in the phosphorylation of Class IIa HDACs by SIK1, transcriptional activation of NFAT2, which positively regulates the expression of the ANP and MHC genes. Thus, the development of SIK1 activators is needed so as to promote the expression of the SIK1 network during the early stages of AKI. The use of physiologic activators such as norepinephrine may have similar effects.

Needless to say, SIK1 activation may not always be beneficial. TGF*β* (whose production starts during the early stages AKI) activates SIK1, and in this manner induces EMT. TGF*β* production has the potential to continue for prolonged periods during AKI [61]. Thus, SIK1 inhibitors can presumably be employed to alleviate the long-term deleterious effects of TGF*β*, including EMT and fibrosis.

PGE_2_ is also produced during AKI. PGE_2_ is a known mediator of inflammatory responses. Nevertheless, AKI was aggravated when microsomal Prostaglandin Synthase 2 (i.e., mPGES-2, which is responsible for renal PGE_2_ production) was down-regulated, and this was associated with increased apoptosis [79]. These latter results suggest that PGE_2_ promotes recovery from AKI. This can presumably be explained by a) the PKA-mediated inhibition of SIK1 which occurs as a consequence of the interaction of PGE_2_ with either EP2 or EP4 receptors, as well as b) the Ca^2+^-mediated activation of calcineurin, which results in the dephosphorylation of CRTC1, and its interaction with pCREB in RPT cells. As a consequence, not only does the expression of the *atp1b1* gene increase, as well as the level of the Na,K-ATPase in RPT cells, but in addition, ATF3 expression increases. Such an increase in the expression of ATF3 (observed in vivo when ATF3 is overexpressed in mice by adenoviral mediated gene transfer) has been observed to result in a reduction of ischemia-reperfusion injury [80]. Thus, SIK1 inhibition at least during the latter stages of AKI presumably promotes recovery.

An increase in mitochondrial biogenesis during the initial phases of AKI is expected to cause an increase in reabsorption, as well as a decrease in the level of ROS. As summarized above, the SIK2 isoform has been observed to restrict mitochondrial biogenesis, due to its ability to phosphorylate CRTCs, thereby preventing the interaction of CRTCs with CREB, and reducing transcription of the PGC1-*α* gene. This limitation can be overcome, presumably by the inhibition of the SIK2 isoform per se during the initiation phase of AKI.

However, the cell cycle progression which occurs during subsequent phases of AKI depends upon SIK2. Not only is the p85 *α* subunit of PI3 Kinase is a substrate of SIK2 (PI3K promoting cell cycle entry) [81], but, in addition, SIK2 is required for cell division itself, being a centrosome kinase [76]. Similarly, the SIK3 isoform is required for mitotic exit. These processes would be promoted by targeting either SIK2 or SIK3 via an activating drug.

Studies conducted both in vitro with cultured renal cells as well as in vivo with transgenic animals are needed in order to evaluate whether the different SIK isoforms participate as anticipated in the events occurring during the different phases of AKI. In addition to employing siRNA and CRISPR technology, a number of expression vectors can be employed, including kinase dead SIK K56M [16], as well as SIK phosphomutants which cannot be phosphorylated by either LKB1, CaMK1 or PKA, the mutants being T182A, T322A, or T577A, respectively in the case of SIK1 [12,16,67]. Similar SIK mutants have been employed in studies with the other SIK isoforms [82,83]. In addition to employing the in vitro approach, studies conducted in vivo with mice possessing targeted knockouts should also prove to be invaluable when studying the role of SIKs in AKI. For example, the use of a mouse strain which expresses CRE recombinase under the control of the SGLT2 promoter will permit targeting of specific SIK knockouts to the RPT [84].

## 5. Development of Clinical Kinase Drugs

Protein kinases have emerged as major drug targets, because their functions within signaling networks are deregulated in a number of disease states [85]. Currently, more than 37 kinase inhibitors have been approved for human use, and the number is expected to increase dramatically, because more than 250 kinase inhibitors are undergoing clinical trials [85]. A number of these drugs target the ATP binding site, which has led to concerns of selectivity. This has led to chemoproteomic target screens of a multitude of kinase inhibitors [85]. Although a number of clinical kinase inhibitors have been found to be nonselective, at the same time other inhibitors were found to be extremely selective, including drugs targeting Mitogen Activated Protein Kinase (MAPK) and the Epidermal Growth Factor Receptor (EGFR). In addition, chemoproteomics have found some unexpected, yet promising drug targets, including SIK2.

SIK2 became of interest as a drug target when a small-molecule screening found that a number of kinase inhibitors that enhanced IL-10 production (and inhibited TNF*α* production) by murine bone-marrow-derived dendritic cells (including the FDA approved drugs dasatinib and bosutinib) actually targeted SIK2 [86]. The SIK inhibitor HG-9-91-01 was observed to have similar effects. In order to identify additional SIK2 inhibitors, MALDI-TOF screening assays have been conducted. In such studies, Heap et al. [87] were able to screen for SIK2 inhibitors, while assaying the phosphorylation of CHKtide, a peptide derived from CHK1 protein kinase involved in DNA repair (CHKtide had been previously identified in a kinase screen as being a good SIK substrate). Not only did Heap et al. [87] successfully identify SIK2 inhibitors, but in addition obtained evidence of activators [87]. Using a similar chemoproteomic screening approach, 21 additional SIK2 inhibitors were identified by Klaeger et al. [85]. Further progress is needed in validating the newly identified SIK2 inhibitors and activators, in addition to identifying drugs specifically targeting the other SIK isoforms.
